# Novel Electromagnetic Characterization Methods for New Materials and Structures in Aerospace Platforms

**DOI:** 10.3390/ma15155128

**Published:** 2022-07-23

**Authors:** David Ramos, José Cidrás, Borja Plaza, Carolina Moravec, Antonia de la Torre, Malte Richard Karl Frövel, David Poyatos

**Affiliations:** 1 Radiofrequency Area, National Institute for Aerospace Technology (INTA), 28850 Madrid, Spain; dramsom@inta.es (D.R.); jcidest@inta.es (J.C.); plazagb@inta.es (B.P.); 2 Composite Materials Area, National Institute for Aerospace Technology (INTA), 28850 Madrid, Spain; cmorpen@inta.es (C.M.); torrelm@inta.es (A.d.l.T.); frovelm@inta.es (M.R.K.F.)

**Keywords:** electromagnetic environmental effects, composite materials, carbon fiber composite, shielding effectiveness, electromagnetic characterization, microstrip transmission line, permittivity, permeability, reverberant chamber

## Abstract

The tendency over the last decades in the aerospace industry is to substitute classic metallic materials with new composite materials such as carbon fiber composites (CFC), fiber glass, etc., as well as adding electronic devices to ensure the safety and proper platform operation. Due to this, to protect the aircraft against the Electromagnetic Environmental Effects (E3), it is mandatory to develop accurate electromagnetic (EM) characterization measurement systems to analyze the behavior of new materials and electronic components. In this article, several measurement methods are described to assess the EM behavior of the samples under test: microstrip transmission line for a surface current analysis, free space to obtain intrinsic features of the materials and shielding effectiveness (SE) approaches to figure out how well they isolate from EM fields. The results presented in this work show how the different facilities from the National Institute of Aerospace Technology (INTA) are suitable for such purposes, being capable of measuring a wide variety of materials, depending on the type of test to be carried out.

## 1. Introduction

Over the last decades, modern aerospace platforms incorporate more and more safety critical electronic systems, whose loss or failure would produce a risk of a catastrophic situation of the air or space platform. This can be seen in the current trend in the aerospace industry when it comes to replacing mechanical systems with electronic systems [1]. Taking all this into account, the Electromagnetic Environmental Effects (E3) to which an aircraft or aerospace vehicle may be subjected can therefore cause adverse effects on the electrical and electronic systems on board, potentially affecting their safety in the different maneuvers of flight [2].

Another problem faced by the aerospace industry in the context of E3 has to do with the proliferation over the last few years of new generation composite materials in the design and manufacture of aircraft platforms [3] and especially in the case of Unmanned Aerial Vehicles (UAVs), where the use of metallic materials is becoming less and less common [4]. In this sense, the use of these new materials means lighter weight aircraft or space frames with better structural performance [5]. Among them, 3D printing stands out due to its ease of design and low cost [6] and composite materials due to their low weight while maintaining good rigidity and structural resistance [5]. On the other hand, the behavior of these new materials in the presence of electromagnetic (EM) fields is not always as good as the ones they replace, resulting in the need for a proper EM characterization as demonstrated in the following examples: Compared to the classic metal surfaces used in aircraft [7], carbon fiber composites (CFC) have less effective shielding, so studying this characteristic is essential to preserve the proper functioning of the electronic devices located inside these platforms that operate in High Intensity Radiated Fields (HIRF) environments. Other than that, the EM characteristics of 3D printed materials differ from the initial raw materials as a result of the phase change from liquid to solid state when printed. In addition, the fill density in the final samples could modify properties such as its permittivity or the loss tangent [8], resulting in variations on the final performance of the actual sensors and electronic equipment that use those materials for their implementation.

Therefore, the importance of EM characterization of these new materials and the need to investigate modern and more flexible characterization methods is clear. As a result, many complex facilities able to perform characterization of materials have been developed in recent years [9,10]. The Equipment and Systems Testing Department of the National Institute of Aerospace Technology (INTA), SPAIN, also designs and operates some of these facilities intended to analyze E3 that can damage equipment or electronic systems and characterize materials on aerospace platforms. In recent years, research projects (UAVEMI (TEC2013-48414-C3-1,2,3-R), eSAFE (PID2019-106120RB-C32) or UAVE3 (TEC2016-79214-C3-1,2,3-R) [11]) have been carried out, highlighting the studies of Electromagnetic Compatibility (EMC), Electromagnetic Interference (EMI), High Intensity Radio Frequency (HIRF), electromagnetic susceptibility, Electromagnetic Pulses (EMP), electronic warfare, the direct and indirect effects of lightning impacts (LDE and LIE) or the precipitation of static charges (P-STATIC). Among the projects discussed, this article focuses on the eSAFE project, which deals with the characterization of the new type of materials (CFC materials, fiberglass, 3D printed materials, etc.) and how they influence the shielding effectiveness (SE) and coupled interference in aircraft wiring, as these are key parameters to guarantee the proper functioning of the safety critical electronic devices of aeronautic and space platforms.

Taking into account all of the above, this article focuses on the description and results obtained from some of the measurement methods developed and available in the department when characterizing materials and aerospace structures for these purposes and will be organized as follows:

In Section 2.1, the materials and structures that have been measured for this article will be shown, and in Section 2.2 the measurement methods used will be described. In Section 3, the results obtained will be presented and discussed, and finally in Section 4, the conclusions obtained from this work are given.

## 2. Materials and Methods

### 2.1. Materials

Taking into account everything mentioned in Section 1, the different aeronautical materials and simple structures that have been characterized are explained below, in order to know their behavior against EM fields when used in the aerospace industry. These are, on the one hand, conductive materials (CFC and unions) and, on the other, dielectric materials (3D printing, fibers and thermoforming plastics).

Regarding conductive materials and structures, the most significant ones are used in a UAV called MILANO [12], a Medium Altitude Long Endurance (MALE) Remotely Piloted Aircraft System (RPAS) developed by INTA with a wingspan of 12.5 m, a length of 8.52 m, a height of 1.43 m and almost entirely made out of CFC.

The samples to be studied are from its central fuselage, being composed of an epoxy matrix Cytec MTM^®^ 45-1 [13] with HexTow^®^ IM7 [14] carbon fiber based on a unidirectional style for each ply, with a nominal thickness of a cured ply of 128 μm. Only “RED+3” sample is slightly different because its layers were stuck with another adhesive, MTA^®^ 240 [15].

The main characteristics of the CFC samples with their corresponding codenames are summarized in Table 1. Different versions of these samples are used in this work, from simple laminates to joints, distinguishing two types: fastener and adhesive ones.

In this work, the CFC samples are characterized through their shielding properties, following both a nested reverberant chambers (RC) method and a coaxial line method, but there are several other approaches which can be taken based on parameters such as the dielectric constant or the conductivity. In [16], a unidirectional and quasi-isotropic version of carbon-fiber-reinforced plastic laminates are assessed by means of an impedance analyzer, obtaining their dielectric constants. The relative permittivity and loss tangent are then measured, reporting values higher than the ones from conventional glass-fiber-reinforced plastic laminates, but the authors claim that the polarization dependencies cannot be explained, so they switch to a shielding characterization method. Another study used a unidirectional and quasi-isotropic carbon fiber reinforced polymer, but this time it was based on free space measurements [17]. In this case, the researchers were studying the conductivity. They observed that for the unidirectional laminate, conductivity increases as frequency does when an incident electric perpendicular field is applied, while when the field was parallel, the conductivity was high and constant over the frequency band. On the other hand, the quasi-isotropic laminate behaves the same regardless of the orientation of the electric field, showing great conductivity in the whole frequency band.

A different approach is carried out in [18], where, with the aim of avoiding discontinuities in measurements for unidirectional carbon fiber reinforced polymer, a waveguide-based configuration is proposed. Here, the sample under test is clamped between two waveguide sections, and the complex permittivity is measured, being later compared with reference ideal models. This type of material is also assessed in terms of its conductivity in [19], which is measured following the Four Point Probe Method. Finally, noncontact methods based on Eddy currents can be found in the bibliography [20]. These have the advantage of being nondestructive and are proven to be suitable to obtain bulk conductivity estimations of the material.

However, as all these methodologies are quite difficult to implement and the SE is a key parameter to evaluate the behavior of CFC materials in the presence of E3, the authors focus their research on methodologies to measure this parameter.

Regarding the fastener joint examined in this article, it is composed by two “RED+3” laminates assembled using Cherry Titanium Maxibolt^®^ rivets [21], FSCM 11.815 type. These blind head rivets are especially used in the aerospace industry because they can be hidden in the fuselage, thus not deteriorating the aerodynamics of the platform.

The distance between rows of rivets is 24 mm and 32 mm between the rivets in the same row, and the overlapped area between both “RED+3” laminates is 56 mm. Additionally, the adhesive joint analyzed consists of two “ORANGE” laminates glued together using Hysol^®^ EA9394 [22] based on epoxy resins with cold curing.

**Figure 1:**
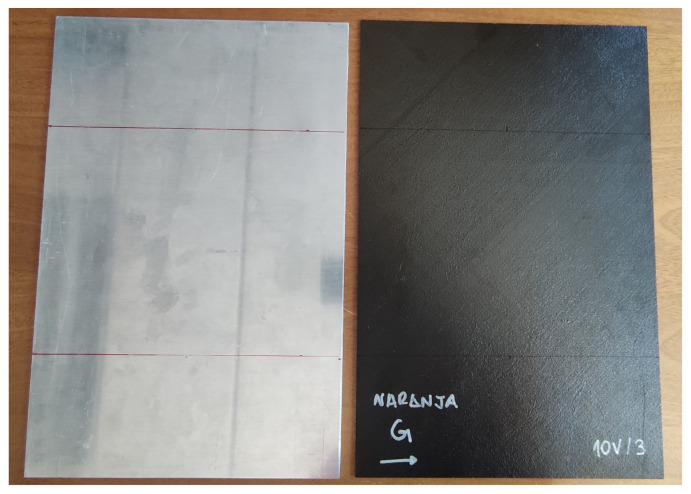
Microstrip test pieces: aluminum (**left**) and CFC sample code named “ORANGE” (**right**).

**Figure 2:**
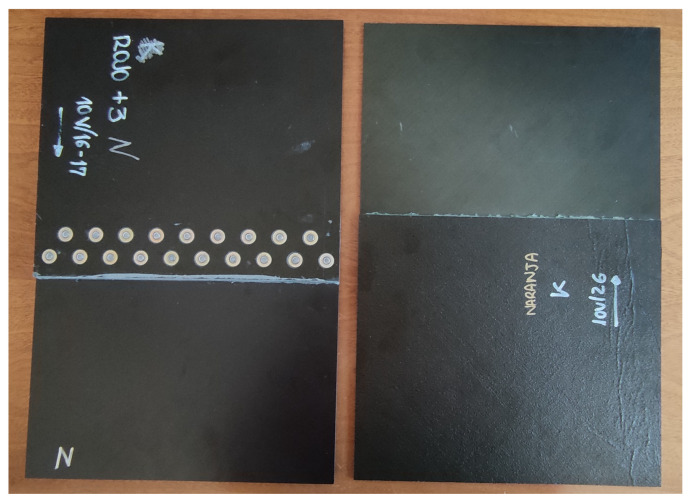
Microstrip test pieces: CFC fastener joint code named “RED+3/RED+3” (**left**) and CFC adhesive joint code named “ORANGE/ORANGE” (**right**).

In Figure 1 and Figure 2, the simple laminates and the two versions of joints studied in this article are shown. The samples from Figure 1, aluminum and CFC “ORANGE”, serve as a reference to evaluate the behavior of the joints.

**Figure 3:**
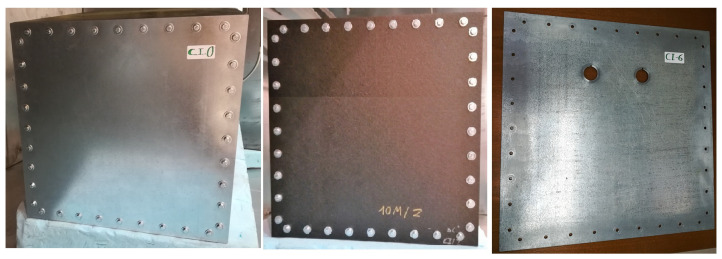
Test cases for the nested RC measurements: CI-0 (**left**); CI-2 (**middle**); and CI-6 (**right**).

In addition, larger samples of CFC and aluminum are used during this work to test SE. These appear in Figure 3, where CI-0 is the reference test case built with aluminum, CI-2 is a CFC sample built with a “BLUE” laminate and CI-6 is an aluminum sheet with circular apertures.

On the other hand, other types of materials related to 3D printing and composites and their use as materials for radomes have been studied. These include low-loss dielectric materials used to protect the antennas that must be transparent to radio frequency (RF) EM energy, both coming from outside and radiated by the antenna itself.

**Figure 4:**
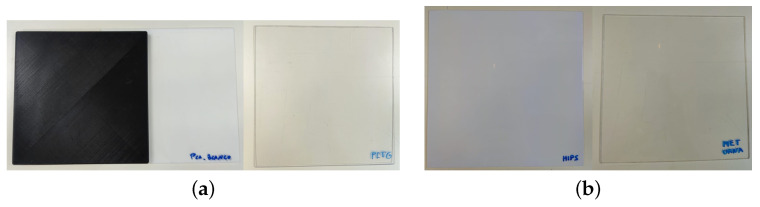
PLA, PETG, HIPS and methacrylate samples: (**a**) PLA white and black (**left**) and PETG (**right**); (**b**) HIPS (**left**) and methacrylate (**right**).

**Figure 5:**
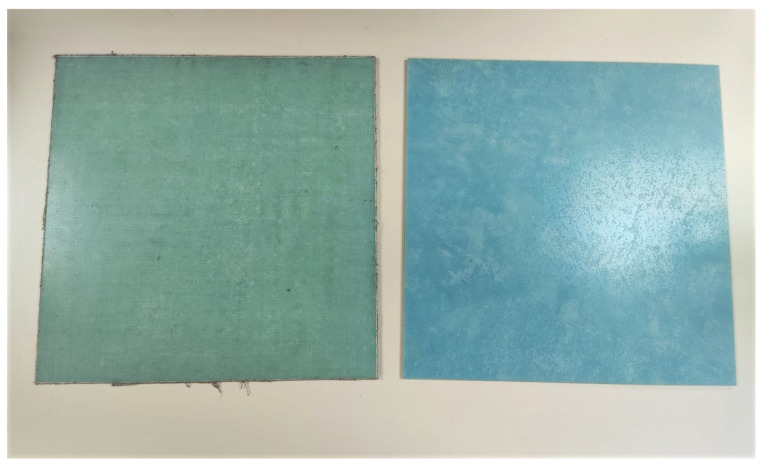
Composite materials of polyethylene fiber (**left**) and quartz (**right**).

In response to this, the following materials have been examined (see Figure 4 and Figure 5). It should be taken into account that the free space technique has been used for their analysis (explained in Section 2.2.2) and that all materials have been mechanized with the same size (the test samples have dimensions of 20 cm × 20 cm) to be able to be used in the measurement bench of the laboratory:

- Methacrylate: Most commonly used plastic with good radome performance in the microwave and millimeter wave ranges. It is moderately priced, easily molded and machinable [23].
- High Impact Polystyrene (HIPS), Polyethylene terephthalate glycol (PETG) and Polylactic acid (PLA): Materials typically used for 3D printing using the Fused Deposition Modeling process (FDM) [24,25].
- FDM process can be used to fabricate complex geometries or internal structures and has been widely applied in many industries, such as the aerospace industry [26].
- Quartz and Polyethylene fibers: These types of fibers are used as reinforcement in prepreg materials. They have excellent electrical properties for a low-loss radome.
- Quartz has a high silica content, resulting in a low dielectric constant and a small loss tangent. It is higher in cost but offers potential lower losses than a polyethylene radome [23].
- Regarding the samples of quartz and polyethylene that will be studied in this article, one sample has been fabricated with Epolam 2025 resin with quartz fibers and another sample has been fabricated with Epolam 2025 resin and polyethylene fiber. For both cases, both samples have dimensions of 30 cm × 30 cm and are 4.4 mm thick. Moreover, Epolam 2025 is a blue-colored epoxy-type resin. This color can be seen in both samples (see Figure 5).

The stacking sequence of each of the samples and their properties is found in Table 2.

Note that the chosen materials are non-magnetic ($\mu_{r}$ = 1) and do not conduct electricity; therefore, only the permittivity will be analyzed, and its expected imaginary part will be very low.

### 2.2. Methods

Currently, there are countless techniques to evaluate the behavior of a material in an EM environment. All these techniques for the EM characterization of materials will depend on the parameters to be extracted. In other words, the use of one technique or another will be defined by the objective of these measurements.

Once the EM parameters or characteristics to be extracted are fixed, a second classification can be made based on different methods to obtain those same parameters.

These different methods to obtain the same EM parameter occur as there is no single measurement method that can electromagnetically distinguish any material or structure in any frequency band largely due to the difficulty of obtaining adequate precision with a single method [27].

That is why there is such a variety of methods for the EM characterization of materials, and the use of each of them will depend on various factors such as: the frequency (or frequency margins) at which the measurement is to be carried out, the required precision, the temperature, the nature of the material, the size of the sample to be tested and whether a destructive test can be carried out in contact with the piece or not.

**Figure 6:**
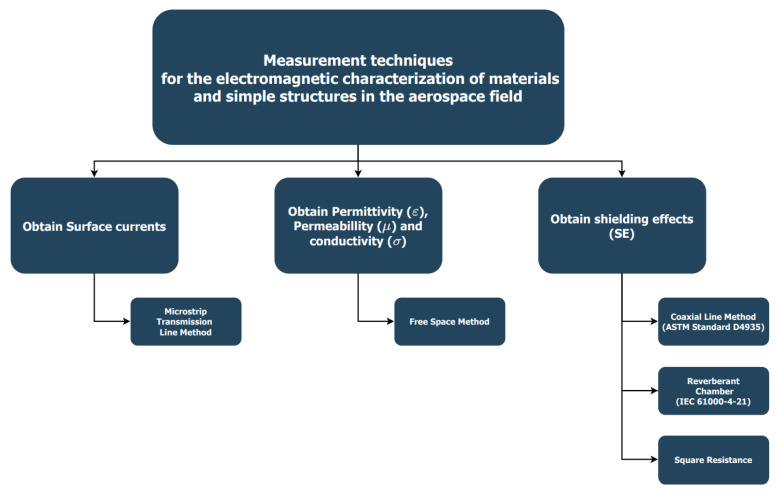
Classification of measurement techniques.

In light of these facts, the following article will discuss different EM measurement techniques whose objective is the study of materials and simple structures in the aerospace industry and in EM environments. All the techniques and methods mentioned have been developed in the Equipment and Systems Testing Department of INTA. In this way, the available techniques will be discussed in terms of obtaining permittivity values of raw materials, obtaining SE and obtaining surface currents. Figure 6 provides a diagram detailing the techniques named above.

On the one hand, there are techniques that yield shielding parameters. These techniques are applicable both to raw materials and to simple structures in which apertures can be studied. In this case, three different methods are named. Two of them are based on a standard that includes measurements in a coaxial line (ASTM Standard D4935 [28]) and measurement in a reverberant chamber (Regulations IEC 61000-4-21 [29]). On the other hand, we have the square resistance method [30].

Moreover, there is the microstrip line technique developed at INTA [31], whose objective is to obtain surface currents. This technique is used for both joints and raw materials such as composite materials with different laminates.

Finally, the techniques related to obtaining permittivity and permeability parameters remain to be discussed. In this case, there are many methods to obtain these parameters such as resonant, perturbation and non-resonant methods.

Non-resonant methods have the advantage of being able to measure in a wide range of frequencies and are based on the determination of the reflection and transmission coefficients of the wave when propagating into the sample to be tested, taking into account the variation of the impedance and speed of this wave.

The Computational and Applied Electromagnetics Laboratory (CAEM-Lab) from INTA has two commercial kits and two test benches focused on the use of non-resonant methods in addition to having developed algorithms for extracting EM parameters through these measurement systems.

Regarding the test benches, there are two measurement facilities that allow measurements to be made in free space. These measurements are the ones on which the study of obtaining permittivities and permeabilities of materials (Figure 6) is focused, which will be developed in the Section 3.2.

As for the commercial kits, there is the DAK (Dielectric Assesment Kit) measurement kit from SPEAG [32] based on the OECP (Open Ended Coaxial Probe) method [33] and the EpsiMu kit from Multiwave [34] based on the transmission line method.

Both kits come with software for extracting the EM parameters. The DAK kit only allows obtaining the permittivity parameter (it only measures reflection parameters), while with the EpsiMu kit it is possible to obtain both permittivity and permeability values [35].

Nevertheless, with the EpsiMu kit, small sample sizes about 13 mm in diameter are needed to fit the cylindrical sample holder. For the DAK kit, sample sizes that can be used in the free space benches can also be used with it. For this reason, in this article, when comparing the measurements between the free space method and one of the kits, only the measurements of the DAK kit will be used.

Once a brief summary of the measurement methods has been made, based on Figure 6, each of the commented techniques will be discussed in more detail.

#### 2.2.1. Microstrip Transmission Line Measurement Method

The purpose of this method is evaluating different types of joints (fastener and adhesive) by measuring their S-parameters and surface currents. S-parameters are analyzed in the frequency band from 10 kHz to 10 MHz, while the frequency band selected to study the surface currents ranges from 10 kHz to 1 MHz. These frequency bands are chosen because according to aeronautical regulations [36], transient and indirect effects can be caused by lightning strikes.

**Figure 7:**
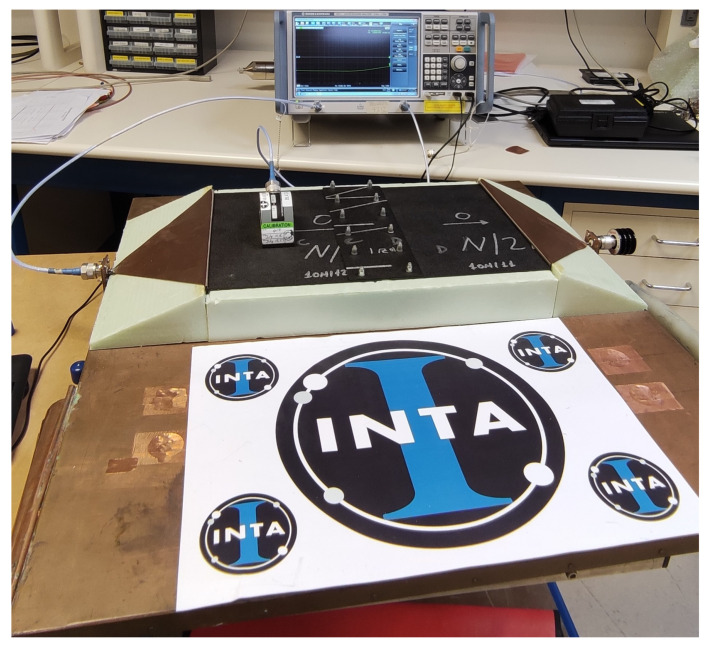
Microstrip transmission line measurement setup.

The test setup is shown in Figure 7, where it can be seen that the equipment used to perform the measurement consists of a Keysight^®^ E5061B vector network analyzer (VNA), the microstrip transmission line and a current probe (employed during the surface currents analysis).

Before we begin, it is mandatory to carry out a calibration of the equipment to obtain accurate values of both S-parameters and surface currents. This calibration eliminates the contribution of the measurement setup. In addition, as the VNA is adjusted to a value of impedance of 50 Ω, the microstrip transmission line is designed to match that value, enhancing the precision of the measurement system. For a proper adaptation of impedances, we take into account that the samples under test are 260 mm wide and that a block of Styrodour^®^ [37] is used to lift them from the ground plane.

Once the equipment is correctly set, the upper conductor of the microstrip line is substituted by the sample to be tested. To calibrate the equipment, a metallic laminate is placed as the upper conductor, with both N-connectors of the VNA plugged to the terminations of the microstrip. With that configuration, the current probe is loaded with an impedance of 50 Ω and put on the metallic sample. Then, the $S_{21}$ parameter is stored. Once this is performed, the 50 Ω load is connected to one of the ports of the microstrip, and the corresponding N-connector is now plugged to the probe. Again, the $S_{21}$ parameter is obtained and referenced to that previously measured. The calibration and the measurement processes are explained in detail in [31].

#### 2.2.2. Free Space Measurement Method

The free space method is a widely used and studied technique [38,39]. These methods use antennas to focus energy on the material to be tested, obtaining the reflection and transmission parameters.

It is therefore a non-destructive technique and without contact with the piece. The fact that it does not require contact with the sample also provides other advantages such as the possibility of carrying out measurements at high temperatures or in hostile environments, with heterogeneous samples, multilayer dielectrics or composite materials.

The use of free space measurement methods has become the most common method for characterization in the microwave region due to its ease of use and reasonable accuracy [40]. However, the free space measurement methods have certain disadvantages, for example, problems at certain resonant frequencies due to the thickness of the sample or the low precision in the calculation of the imaginary part of the permittivity in dielectric materials with very low losses.

Specifically, this measurement technique is one of the methods used in the CAEM-Lab from INTA, where there are two types of facilities, (POLYBENCH [8] and BIANCHA [41]), capable of performing measurements in this type of method and from which different algorithms for extracting EM permittivity and permeability values developed at INTA can be applied.

**Figure 8:**
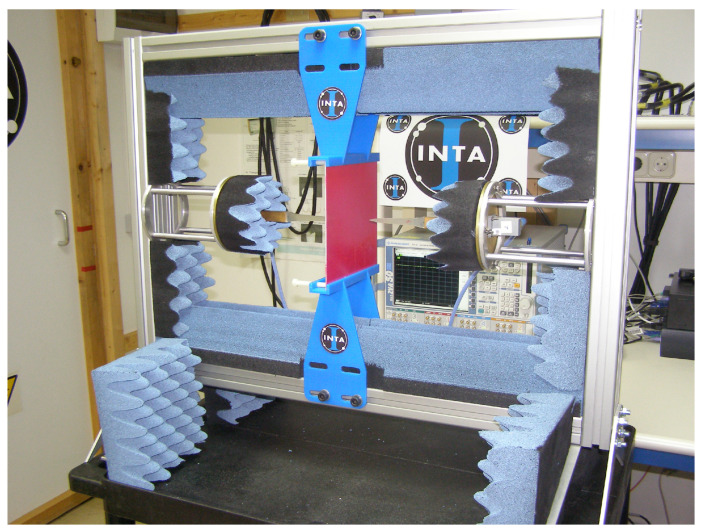
POLYBENCH. Measurement bench with high-precision polyrod antennas.

The POLYBENCH (see Figure 8) is a high-precision bench made up of a radiating element (polyrod antennas) and a rigid structure (providing robustness and precision), covered with absorbent material to reduce the noise level of the environment.

The test bench is connected to a Rhode and Schwarz ZVA 50 4-port VNA using coaxial cables. The measurements of the scattering parameters (S-parameters), which provide information on the transmission and reflection coefficients, are extracted using a custom software self-developed by the CAEM-Lab.

**Figure 9:**
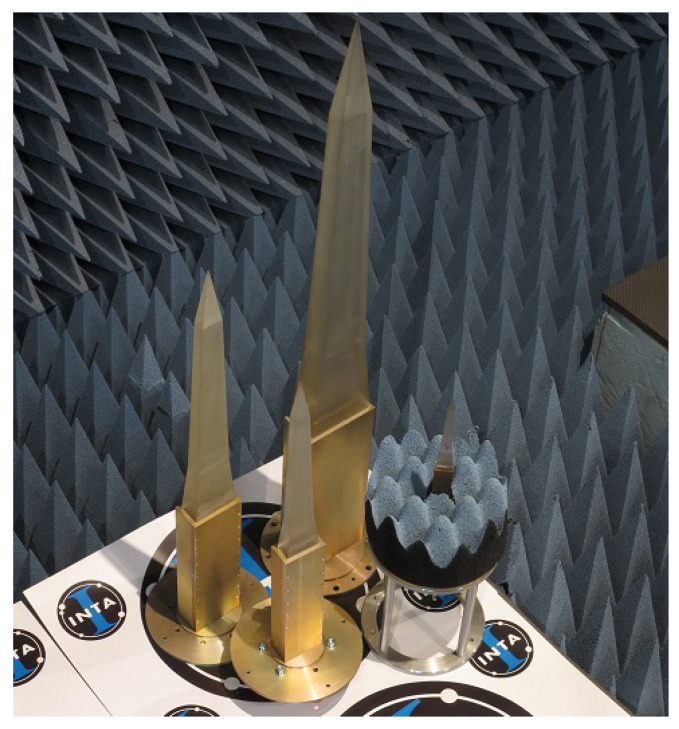
2.6 GHz to 18 GHz polyrod antennas.

The fundamental characteristic of polyrod antennas is the sharp pyramidal shape (whose material is rexolite) on the end of the metal waveguide (see Figure 9). It allows the emitted electric field to be focused in the direction of propagation, concentrating it in the central part of the tested material. This avoids diffraction errors at the edges, provides higher measurement accuracy and also allows the use of smaller samples [8].

This fact favors the precision in obtaining the values of the characteristic transmission and reflection coefficients of the material under test and therefore increases the calculation of the permittivity and permeability values of the developed algorithms.

**Figure 10:**
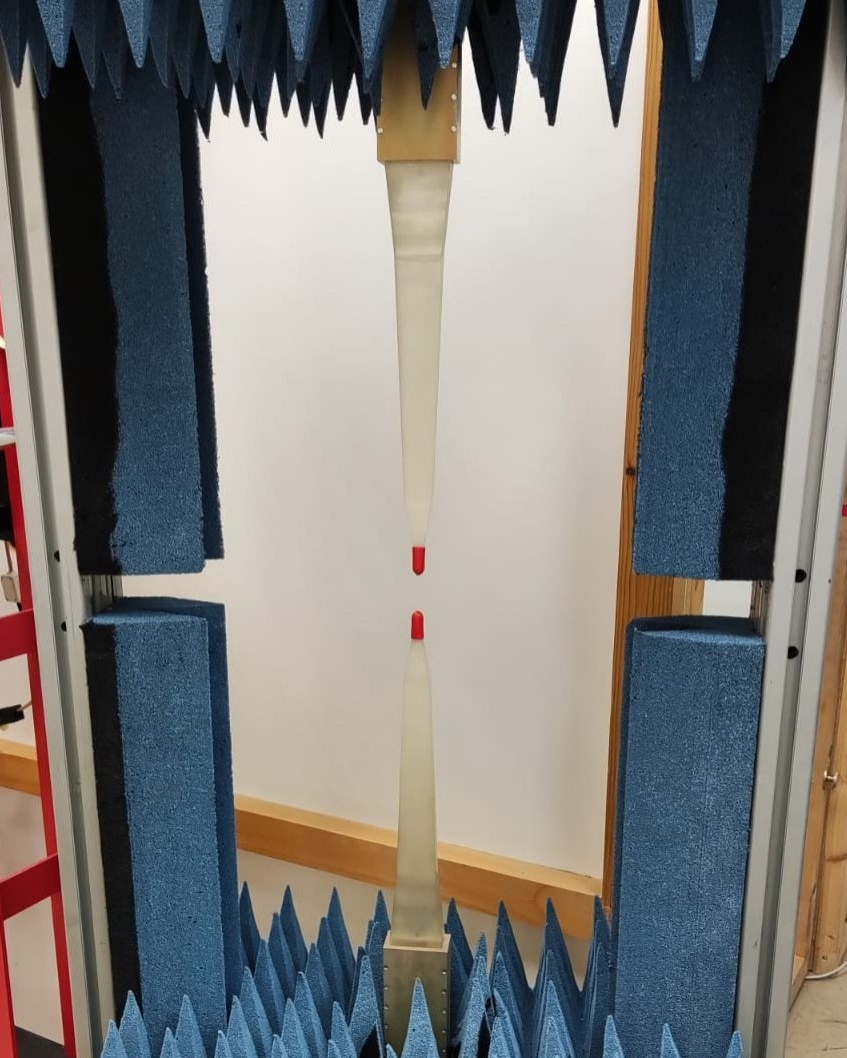
Vertical measurement bench with high-precision polyrod antennas.

The size of the antennas depends on the operating frequency of each of them. In the case of POLYBENCH, there is a measurement range from 8.2 GHz to 40 GHz; however, there are larger antennas that allow measurements at frequencies from 2.6 GHz to 8.2 GHz. Due to their size, these latter antennas cannot be used in this bench, although a system similar to the POLYBENCH is available vertically (see Figure 10) where these antennas can be used.

**Figure 11:**
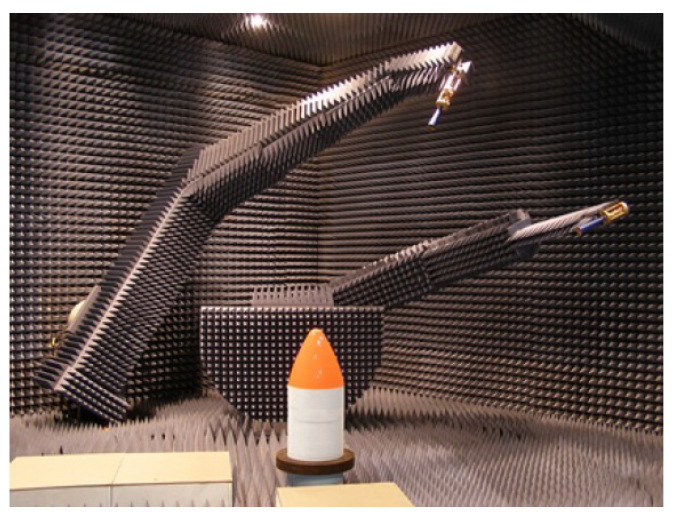
BIANCHA (Bistatic ANechoic CHAmber).

It must be taken into account that BIANCHA (see Figure 11) is designed not only to obtain reflection and transmission values, but also for other types of EM measurements, such as radiation and scattering, with an estimated precision of 0.5 dB [8].

That is why, for the case of this study, in which only mono-static reflection and transmission measurements are required to obtain the permittivity and permeability values of the materials, the use of this facility is not needed, especially since there is another type of measurement bench, such as the POLYBENCH, with which a greater range of frequencies can be obtained with an accuracy in the reflection and transmission parameters much better than BIANCHA.

As mentioned above, the test is based on obtaining the values of the reflection and transmission coefficients of a material, and these are obtained from the measured S-parameters which are obtained on the test bench by means of a VNA.

**Figure 12:**
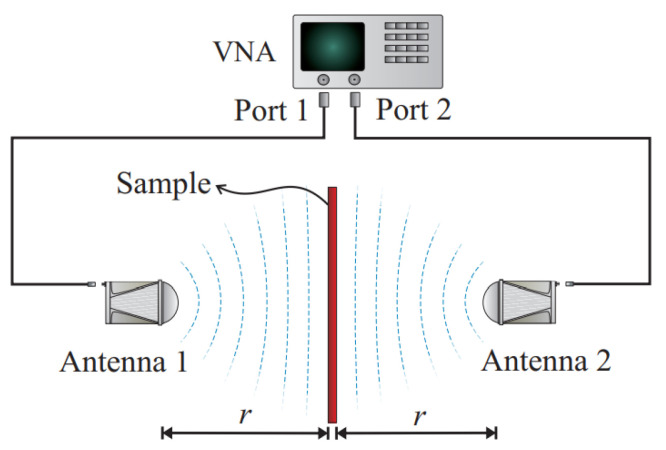
Free Space measurement method representation for obtaining transmission and reflection parameters. The sample is placed between two antennas, which are connected to the ports of the VNA [42].

In our study, since there are two opposite antennas, it will be interesting to obtain the values of the parameters $S_{11}$ (emitting the signal through port 1 and receiving it in the same port) and $S_{21}$ (emission of the electromagnetic signal through port 1 and receiving it in port 2), see Figure 12.

The experimental procedure to carry out the measurements is as follows:

1. It will be necessary to make a total of three measurements for each material to be tested and for each of the parameters S: first the measurement of the material itself arranged as it appears in Figure 8, a second measurement where the free space (air) is measured, and the third measurement that corresponds to a metal plate (both used as reference).
2. Once these three measurements have been made, using the *S*-parameters ($S_{11}$ and $S_{21}$) for each of them, it is possible to obtain the transmission and reflection coefficients sought.
3. However, the measured *S*-parameters are influenced by multipath propagation, unwanted reflections and other interference, especially in a non-anechoic environment. As a consequence, a gating of the signal in the time domain is performed. In this way, we will have the parameter $S_{21}$ just after the material and the parameter $S_{11}$ before the material. This process can be carried out using the VNA, but it is of vital importance to determine the test window correctly to avoid disturbances in the values obtained for reflection and transmission.
4. Once the gating has been applied, obtaining the reflection and transmission from the *S*-parameters, respectively, is as follows:

(1) $$\Gamma =-\frac{S_{11_{material}}-S_{11_{air}}}{S_{11_{metalplate}}-S_{11_{air}}}$$

(2) $$T=\left(\frac{S_{21_{material}}-S_{21_{metalplate}}}{S_{21_{air}}-S_{21_{metalplate}}}\right)e^{-i2\pi d/\lambda_{0}}$$

where $\lambda_{0}$ is the wavelength in air, and *d* is the thickness of the sample.

#### 2.2.3. Shielding Effectiveness Measurement Method

There exist several methods to study SE. The choice depends on several factors, such as the shape of the material, the structure where the material is placed, etc. In this work, the focus is set on three different approaches:

- ASTM Standard D4935 [28];
- Regulations IEC 61000-4-21 [29];
- DC Square resistance (ohmic resistance) [30].

**Figure 13:**
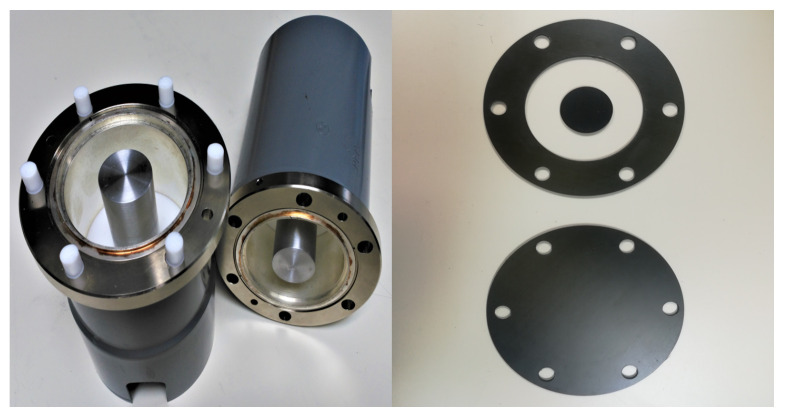
Two 1/8 EIA to N female adapters used as sample holder (**left**), reference (**upper right**) and load (**lower right**) samples.

The ASTM Standard is best suited for raw flat materials. It is a guideline to measure SE of flat solid samples confined within a coaxial line under the influence of EM fields. The standard defines the limitation in frequency of this technique, which covers from 30 MHz to 1.5 GHz, as well as the sample holder that must be used to carry out the tests, which is an enlarged, coaxial transmission line with special taper sections and notched matching grooves to maintain a characteristic impedance of 50 Ω throughout the holder. A commercially available version from SPINNER [43] of these sample holders can be seen in Figure 13, along with the reference and load samples necessary to perform the tests.

**Figure 14:**
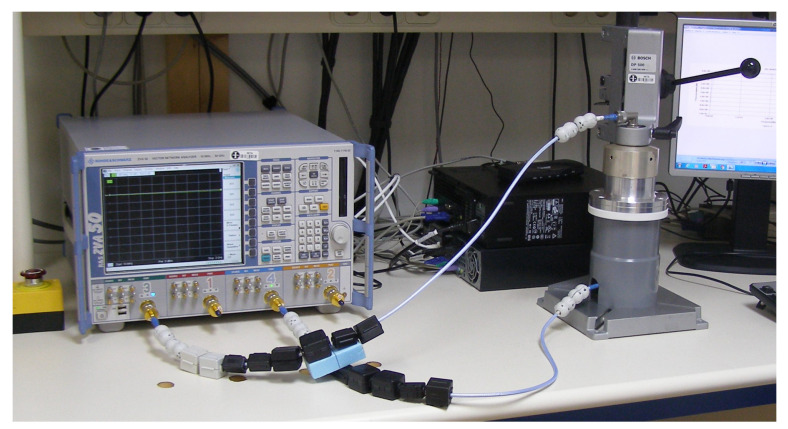
Measurement setup according to ASTM D4935 standard.

To perform the measurements, a VNA is used. Ports 1 and 2 are directly connected to the sample holders through coaxial cables as shown in Figure 14.

By doing so, the VNA is capable of obtaining the *S*-parameters related to the material tested. With these data, the SE calculation is performed according to the expression (3):

(3) $$SE=20log_{10}\left(\frac{|S_{21,R}|}{|S_{21,L}|}\right),$$

where $S_{21,R}$ represents the $S_{21}$ parameter from the reference, and the $S_{21,L}$ corresponds with the $S_{21}$ from the load (see Figure 13, lower right). When performing the measurement, the process to follow is: first obtain the $S_{21}$ parameter of the reference level by setting the reference sample, and then, simply substitute it for the load sample and repeat the measurement.

Both of them, reference and load, are made out of the same type of material. The only difference is that, as shown in Figure 13 (upper right), the load covers the whole space inside the coaxial line, providing the lowest $S_{21}$ values in the specified band of frequency, while the reference does not, producing the highest $S_{21}$ values.

However, when the test sample to be measured is not a simple piece of material, but a more complex structure, such as joints, apertures, etc., it is necessary to apply the IEC 61000-4-21 regulations. There, it is described how to use a reverberant chamber to perform measurements. In addition, this method offers an extended frequency range (up to 40 GHz) compared to the ASTM. If the samples under test are incorporated in larger structures, the “nested chambers” procedure must be applied. To do so, the RF source must be connected to the main chamber. Then, the measurement equipment is connected to the receiver antenna which is placed inside the inner chamber, and the main chamber reference antenna is connected to the monitoring equipment. Once this is performed, the tuners located inside the main chamber start to move, and the first test frequency is injected at a fixed input power. Now, the maximum readings from the chamber monitoring equipment and the inner chamber are recorded. This last step is repeated for all the test frequencies. All these steps are further explained in annex H from [29].

**Figure 15:**
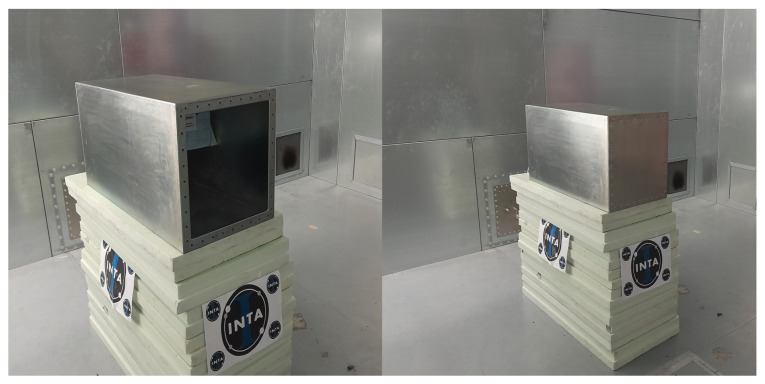
Nested RCs: reference configuration (**left**) and sample under test (**right**).

The SE value is obtained as shown in (4):

(4) $$SE=10log_{10}\left(\frac{P_{REF}}{P_{EUT}}\right),$$

where $P_{REF}$ is the power coupled to the reference antenna (which is located in the larger RC), and $P_{EUT}$ is the power coupled to the equipment under test. This implies that to calculate the SE value, two different measurements must be taken. In both cases, the enclosure is already placed inside the larger RC. The difference is whether the sample under test is not assembled (reference configuration) or if it is. In Figure 15, these two measurements are shown.

As the aforementioned methods present certain limitations, there is one more alternative to carry out SE measurements, which is using the DC square resistance [44], also called ohmic resistance, of the materials to be analyzed. Its main advantage is its simplicity. For this method, the SE value is obtained through the measurement of the DC square resistance and the effective thickness of the sample.

**Figure 16:**
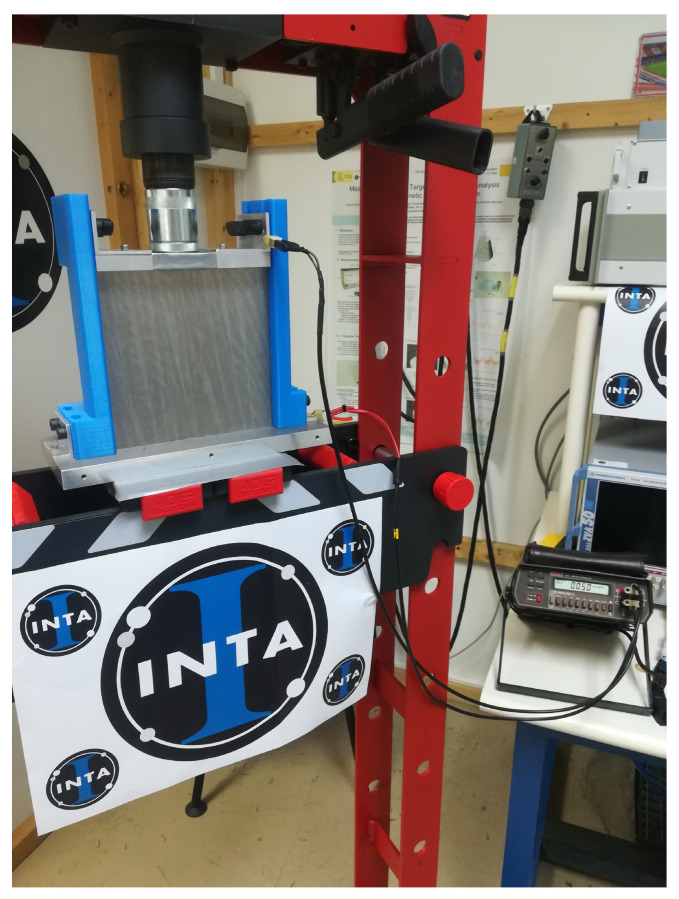
DC square resistance test setup.

This test is carried out using a sample holder, a micro-ohmeter and a pneumatic press. In this case, the only thing to do is to place the sample under test in the sample holder, apply pressure on the sample holder to guarantee a very low impedance ohmic contact between the sample and the holder and record the value registered by the micro-ohmeter. The sample holder consists of two structures made of a dielectric polymeric material between which the material is placed, as shown in Figure 16. The press is the commercial model MEGA PRC-20 [45] and the micro-ohmeter is the model 580 from Keithley [46]. Once the resistance of the material is measured, SE values are obtained through the formulation from [44].

All these methods to obtain SE presented in this section are explained with further detail in [30].

## 3. Results and Discussion

In this section, the results corresponding to the microstrip transmission line, free space and the three different SE measurement approaches are shown and explained in detail.

### 3.1. Microstrip Transmission Line Measurement Method

The test cases analyzed for this method are studied in terms of *S*-parameters and surface current.

**Figure 17:**
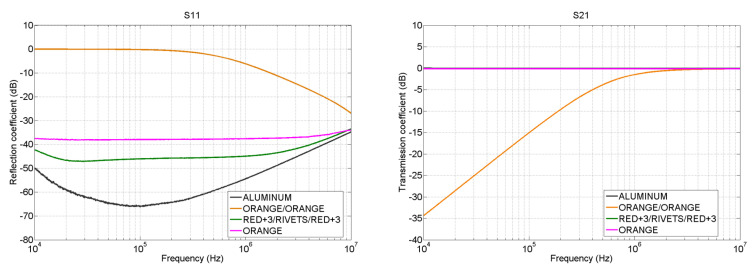
Microstrip S-parameter measurement: $S_{11}$ (**left**) and $S_{21}$ (**right**).

The results corresponding to the *S*-parameters are shown in Figure 17. There, the $S_{11}$ and $S_{21}$ parameters are depicted. The *S*-parameters inform about the transfer of power which is ocurring from one end of the sample under test to the other. In the case of the $S_{11}$, the higher the value, the higher the reflected power to port 1 of the VNA is. On the other hand, $S_{21}$ refers to the power transfered from port 1 to port 2 of the VNA. The higher this value is, the more power is reaching port 2 from port 1. Having this in mind, several conclusions can be obtained. In the image corresponding to $S_{11}$ parameter, it can be seen that the aluminum is the sample which is reflecting less power back to port 1, followed by the fastener joint “RED+3/RED+3” and the CFC sample “ORANGE”, while the worst in terms of transmission of power is the adhesive joint “ORANGE/ORANGE”. The $S_{11}$ and $S_{21}$ graphs are complementary. Since the reflected power by the aluminum, “ORANGE/ORANGE” and “RED+3/RED+3” is really low, when observing the $S_{21}$ graph, all of them are shown as good transmitters of power. However, the opposite happens with the adhesive joint.

**Figure 18:**
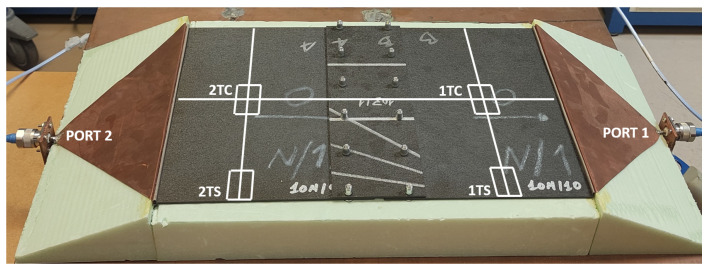
Microstrip surface current measurement: analysis sections.

Before commenting on the surface currents results, it must be explained how they were carried out. The currents might not flow evenly through the whole surface of the samples, and, in the case of the joints, there may be differences depending on which side of the joint is being measured. That is why we measured several positions along the surface of the sample with the current probe. The results can be seen in Figure 18. For both halves of the sample, the measurements were taken in the central section (TC) and one of the sides (TS).

**Figure 19:**
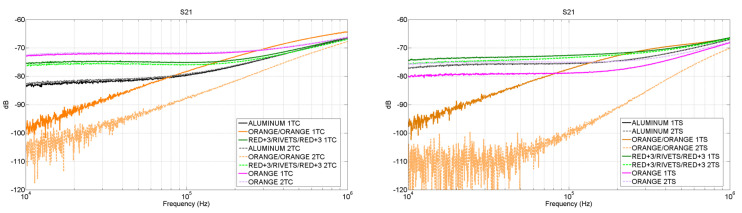
Microstrip surface current measurement: 1TC/2TC central sections of the sample (**left**) and 1TS/2TS side sections of the sample (**right**).

Once this is clear, the results corresponding to the surface current measurements are shown in Figure 19. The graph on the left represents the results from the central sections of the sample, while the graph on the right corresponds with the results obtained from the sides.

Focusing on the behavior of the aluminum first, it can be seen that, as expected, it does not matter which half of the sample is measured because the distribution of currents remains constant. However, it does change depending on whether the current value is taken on the central section or on the side section. Higher values of current are found on the side sections. This can be explained because a finite metallic laminate has an edge effect which creates more suitable paths for the transmission of the currents. Compared with the aluminum, the CFC laminate “ORANGE” shows a different behavior. In this case, the surface currents flow better through the central sections than the side ones. Due to this, the surface current in the central sections is higher for the “ORANGE” sample, but in the side sections, the aluminum shows a better performance.

Regarding the joints, the fastener “RED+3/RED+3” shows higher surface current values than the aluminum in both sections of study, central and side. In addition, the currents flowing on its surface are not affected either by the joint or by the section analyzed, presenting similar values on both halves of the sample and on the central and side section of the laminate. This can be explained because for this CFC sample the distribution of conductive filaments can be considered homogeneous at a macroscopic level. On the other hand, the surface current from the adhesive joint “ORANGE/ORANGE” presents transmission losses across the joint in both central and side sections.

### 3.2. Free Space Measurement Method

Once the reflection and transmission values have been obtained, the values of the EM parameters such as permittivity and permeability remain to be determined. As mentioned above, the CAEM-Lab has developed various algorithms that are based on obtaining these parameters from reflection and transmission measurements following the equations:

(5) $$T=\frac{4\sqrt{\frac{\mu_{2}}{\varepsilon_{2}}}e^{-jwd\sqrt{\varepsilon_{0}\mu_{0}\varepsilon_{2}\mu_{2}}}}{{\left(1+\sqrt{\frac{\mu_{2}}{\varepsilon_{2}}}\right)}^{2}-e^{-2jwd\sqrt{\varepsilon_{0}\mu_{0}\varepsilon_{2}\mu_{2}}}{\left(1-\sqrt{\frac{\mu_{2}}{\varepsilon_{2}}}\right)}^{2}}$$

(6) $$\Gamma =\frac{\left(\frac{\mu_{2}}{\varepsilon_{2}}-1\right)\left(1-e^{-2jwd\sqrt{\varepsilon_{0}\mu_{0}\varepsilon_{2}\mu_{2}}}\right)}{{\left(1+\sqrt{\frac{\mu_{2}}{\varepsilon_{2}}}\right)}^{2}-e^{-2jwd\sqrt{\varepsilon_{0}\mu_{0}\varepsilon_{2}\mu_{2}}}{\left(1-\sqrt{\frac{\mu_{2}}{\varepsilon_{2}}}\right)}^{2}}$$

where $\varepsilon_{2}$ and $\mu_{2}$ are, respectively, the permittivity and permeability of the material under test.

Once the equations that link the measurements with the values or parameters that are to be obtained have been seen, some of the results obtained through the measurements in the laboratory will be now presented.

In this case, to obtain the EM parameters, the Particle Swarm Optimization (PSO) method, described in [47], has been used. It is a genetic algorithm-style optimizer that is based on the application of a model present in nature to solve engineering problems. In the case study, this method has been implemented using Matlab, whose search for complex permittivity and permeability parameters is based on the minimization of an error function in which the measured reflection and transmission values and the theoretical values of Equations (5) and (6) are subtracted.

This error function has been implemented in different ways to study which is the most appropriate in the search for the solution. In this way, error functions have been implemented based on pure complex numbers, on modules and phase or taking into account arithmetic means or variances. The results obtained correspond to the most stable solution obtained with these different error functions.

#### 3.2.1. Validation with Teflon and Eccostock Hick

For the first case, the values of reference materials will be represented to show the viability of the algorithm used.

One of the materials to be used is Teflon since is commonly used as a reference when it comes to electromagnetically characterizing materials. On the other hand, it is a dielectric material with a real permittivity value of 2.1, stable in a wide range of frequencies [48].

The measurements of three reference materials with known EM characteristics will also be represented. These materials are called Eccostock Hick K = 3, K = 5 and K = 7, from Laird Technologies [49], with real permittivity values of 3, 5 and 7, respectively.

**Figure 20:**
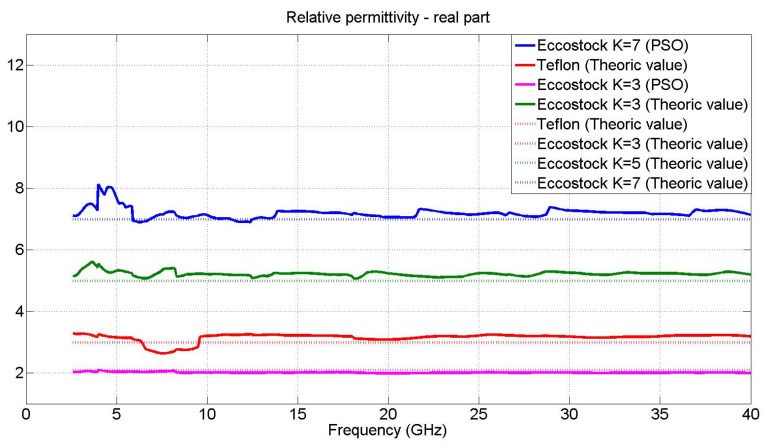
Comparison, from 2.6 to 40 GHz, between the values of the real part of the permittivity of Teflon and Eccostock (K = 3, K = 5 and K = 7), PSO and Theoric values.

The measures of the mentioned materials can be observed in Figure 20.

As can be seen, adequate values are obtained according to the theoretical value of the reference materials (see Figure 20); therefore, the values obtained from the other materials mentioned in Section 2.1 of this article are represented below.

#### 3.2.2. Quartz and Polyethylene Fibers

Two samples of prepreg material are available for these measurements (described in the Section 2.1).

It is estimated that the value of the real part of the permittivity for these materials, based on the type of fiber and resin used, is 2.8 in the case of polyethylene fiber and 3.1 in the case of quartz fiber (see Table 2) [23].

**Figure 21:**
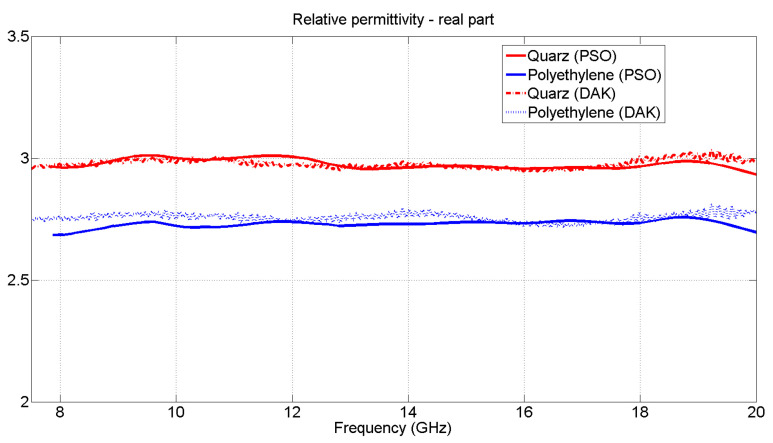
Comparison, from 8 to 20 GHz, between the values of the real part of the permittivity of quartz fiber (in red) and polyethylene (in blue), PSO and OECP (DAK Kit) methods.

The results obtained from the measurements made are collected in Figure 21, where both the values obtained with the DAK Kit and with the PSO algorithm are similar to those found in the literature.

#### 3.2.3. Methacrylate, HIPS, PETG, PLA

In this case, these four materials are in 20 cm × 20 cm samples (see Figure 4) whose thicknesses and theoretical permittivity averaged values (measured with the DAK kit) are listed, along with the values obtained by the PSO algorithm in Table 3.

**Figure 22:**
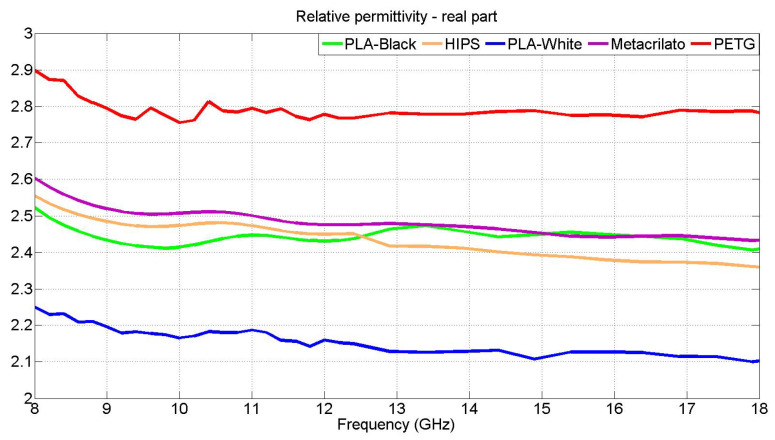
Comparison, from 8.5 to 20 GHz, between the values of the real part of the permittivity of methacrylate, HIPS, PETG and PLA, obtained by PSO method.

The results of the permittivity values of each of the studied samples in the frequency range between 8.5 and 20 GHz appear in Figure 22.

As can be seen, values are very close to those obtained with the DAK Kit.

### 3.3. Shielding Effectiveness Measurement Method

As stated in Section 2.2.3, in this work, three different approaches to perform SE measurements are considered. The results are presented in the same order followed when explaining the methods: ASTM, RC and finally, DC square resistance.

Regarding the ASTM standard measurements, CFC samples code named as “BLUE”, “ORANGE” and “RED” whose characteristics can be seen in Table 1 were measured. These samples were selected because they correspond to laminates from the MILANO UAV.

**Figure 23:**
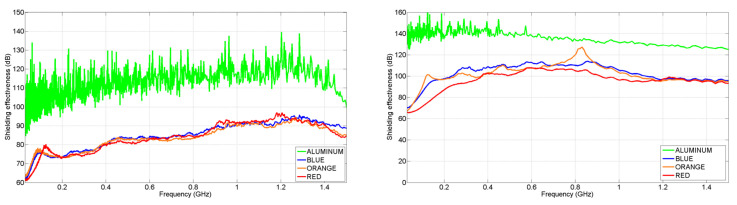
Shielding effectiveness measurement according to ASTM D4935 standard: before upgrading the sample holder (**left**) and after upgrading the sample holder (**right**).

The results are shown in Figure 23. The first set of measurements was made using the commercial sample holder from SPINNER, which corresponds to the graph on the left. There, it can be seen that the aluminum presents the highest SE value, acting as the reference value and that all the CFC samples show high SE values as well independent of their number of plies. However, in this case, there are two issues that make the accuracy of the measurements suspicious. First, the SE of the aluminum starts to decrease from 1.2 GHz, and second, SE values of some samples are too close to the background noise level because, according to Section 8.5 in [28], they must be at least 10 dB above.

**Figure 24:**
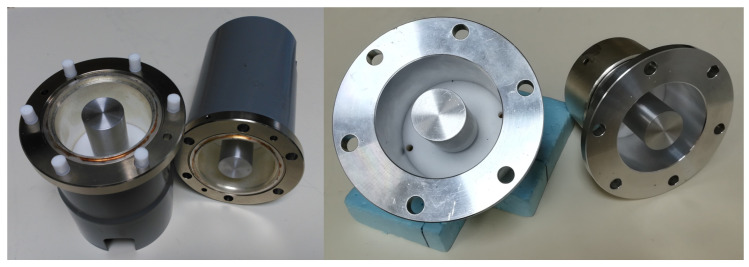
Sample holder improvement to perform measurements according to ASTM D4935: commercial sample holder (**left**) and upgraded sample holder (**right**).

Due to this, a new sample holder was built in INTA. In this new version, the surfaces were polished to eliminate a ring discontinuity existing in the commercial one, as shown in Figure 24.

The results obtained are represented in the graph on the right from Figure 23. With this improvement, the SE of the aluminum does not decay at 1.2 GHz as happened before. In addition, there is a noticeable change in the SE values of the CFC samples, increasing its performance up to 20 dB with respect to the former results, depending on the frequency.

The second method explained is based on measurements using nested RC. This approach applies when the sample under test is included in another structure, which must be located inside a larger RC. In this case, the material under test is assembled in the inner RC as shown previously in Figure 15. For these measurements, the test cases are the ones depicted in Figure 3. They are screwed on one of the sides of the smaller RC using a metallic gasket which increases the contact of the joints, resulting in a better continuity between the material and the chamber.

**Figure 25:**
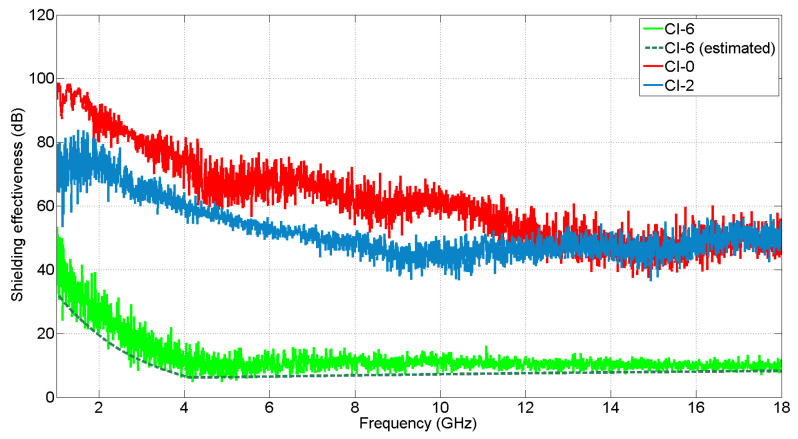
Shielding effectiveness measurement according to IEC 61000-4-21 standard.

CI-0 is a metallic panel, which means that it should offer the highest value of SE, which translates into less RF energy being leaked inside the enclosure. Then, CI-2 is a CFC “BLUE” panel and finally, CI-6 is a version of CI-0 with circular apertures. The results in the frequency band from 1 to 18 GHz are presented in Figure 25. First, the correct functioning of the chamber must be assessed. That is why the test case CI-6 is compared with the theoretical results according to the formulation in [50], obtaining a similar outcome. Once this is obtained, the rest of the results can be evaluated. CI-0 agrees with the theoretical behavior, showing the highest SE value from 1 to 12 GHz. From there, both CI-0 and its CFC version CI-1, show analogous values in the band from 12 GHz to 18 GHz. This effect can be explained because, at higher frequencies, the CFC panel start to behave like a metallic one in terms of conductivity. However, it is something to be studied in future measurements to check if cable leakages or other undesirable effects could be interfering. Finally, it is CI-6, the panel with the circular apertures, the one showing the worst result in terms of SE, as the RF energy flows inside the structure through them.

Apart from the test cases presented in this work, there are other samples to be tested in future studies. With them, it could be possible to analyze with further detail how gaps of different shapes in the surface of the laminates affect the total SE of the enclosure.

The third and last method uses the DC square resistance. As explained before, it is based on the formulation described in [44], which allows us to obtain *SE* values from square resistance results. This approach requires simpler equipment than the other two methods explained, but it is limited in frequency up to 30 MHz. The samples selected for these measurements were two 20 cm × 20 cm CFC panels: “BLUE” and “RED”. Furthermore, the residual resistance of the assembly is analyzed using an aluminum laminate.

The results for this method are summed up in Table 4. Due to the high conductivity of aluminum, its resistance value is very low, and according to the theoretical calculations, its *SE* value tends to infinite. That is why it appears as “not applicable” in the table. For the CFC samples, an unequivocal relation between the number of plies oriented in the transmission direction and the square resistance resulting value can be observed.

## 4. Conclusions

The tendency over the last decades in the aerospace industry is replacing the classic metallic materials with new advanced ones, such as 3D printed and composite materials. This is due to an easier design phase together with lower cost and weight compared to metallic pieces. In addition, there is a need to provide modern air and space platforms with smart skin capabilities, adding different elements to the fuselage such as embedded sensors for a better monitoring of the platform or frequency selective surfaces (FSS), to act as physical filters to certain RF emissions. As a consequence of all these changes, the importance of a correct EM characterization of new materials and components rises. It is mandatory to develop proper techniques to assess their performance.

In this context, INTA has designed and operated different and flexible methods such as surface currents analysis and extraction of intrinsic characteristics of materials, including permittivity, permeability and conductivity, as well as studying SE of materials through three approaches. Apart from the results presented in this article, all these methods can be used with a wider variety of materials. The microstrip setup can be employed with different combinations of fastener and adhesive joints. Free space measurements provide great flexibility due to the design of the measurement systems. Further research on this topic would involve analyzing magnetic materials or structures such as FSS. Finally, regarding SE measurements, a future line of research relies on gaining knowledge about how different apertures affect the resulting SE value of an enclosure.

## Figures and Tables

**Table 1:** MILANO UAV laminates.

Material	Stacking Sequence	Number of Plies
BLUE	[+45/−45/−45/0/+45/90/+45/0/−45/ −45/0/+45/90/+45/0/−45/−45/+45]	18
ORANGE	[+45/−45/+45/0/+45/90/+45/0/−45 /−45/0/+45/90/+45/0/+45/−45/+45]	18
RED	[+45/−45/0/−45/+45/90/ 90/+45/−45/0/−45/+45]	12
RED+3	[45/−45/0/−45/45/90]${}_{S}$ [45/−45/0/90/0/−45/0/0/45/0/90/0]${}_{S}$	36

**Table 2:** Prepeg of quartz and polyethylene fibers, laminates properties.

Material	Stacking Sequence	Number of Plies	Resin	Permittivity Theoric Real Part [23]
Polyethylene	[(0/90)]${}_{34}$	34	Epoxy	2.8
Quartz	[(0/90)]${}_{14}$	34	Epoxy	3.1

**Table 3:** Methacrylate, HIPS, PETG and PLA samples properties.

Material	Thickness (mm)	Mean Value Permittivity—Real Part (Measure with DAK Kit) [1–20 GHz]	Mean Value Permittivity—Real Part (PSO Algorithm) [8.5–20 GHz]
Methacrylate	2.93	2.56	2.493
HIPS	1.93	2.41	2.437
PETG	1.97	2.77	2.79
PLA-white	3.2	2.14	2.1483
PLA-black	10.2	2.42	2.442

**Table 4:** Shielding effectiveness measurements using DC square resistance technique.

Name	Square Resistance	Estimated SE for f < 30 MHz
Aluminum	0.5 mΩ	Not applicable
RED	99.6 mΩ	75.2 dB
BLUE	46.4 mΩ	78.7 dB

## Data Availability

Not applicable.

## Abbreviations

The following abbreviations are used in this manuscript:

EM	Electromagnetic
CFC	Carbon Fiber Composites
HIRF	High Intensity Radiated Fields
UAV	Unmanned Aerial Vehicle
MALE	Medium Altitude Long Endurance
RPAS	Remotely Piloted Aircraft System
INTA	National Institute of Aerospace Technology
RF	Radiofrequency
HIPS	High Impact Polystyrene
PETG	Polyethylene Terephthalate Glycol
PLA	Polylactic Acid
FDM	Fused Deposition Modeling
SE	Shielding Effectiveness
CAEM-Lab	Laboratory of Computational and Applied Electromagnetism
DAK	Dielectric Assessment Kit
OECP	Open Ended Coaxial Probe
VNA	Vector Network Analyzer
BIANCHA	Bistatic Anechoic Chamber
RC	Reverberant Chamber
LUF	Lowest Usable Frequency
PSO	Particle Swarm Optimization
FSS	Frequency Selective Surface
